# Homosexual Behavior in Female Mountain Gorillas: Reflection of Dominance, Affiliation, Reconciliation or Arousal?

**DOI:** 10.1371/journal.pone.0154185

**Published:** 2016-05-11

**Authors:** Cyril C. Grueter, Tara S. Stoinski

**Affiliations:** 1 School of Anatomy, Physiology and Human Biology, The University of Western Australia, Crawley (Perth), WA, Australia; 2 The Dian Fossey Gorilla Fund International, SE Atlanta, Georgia, United States of America; University of Pisa, ITALY

## Abstract

Humans are unique among primates for not only engaging in same-sex sexual acts, but also forming homosexual pair bonds. To shed light on the evolutionary origins of homosexuality, data on the occurrence and contexts of same-sex behavior from nonhuman primates may be of particular significance. Homosexual behavior involving females is poorly researched in most primate taxa, exceptions being Japanese macaques, rhesus macaques, Hanuman langurs and bonobos. We present data on homosexual behavior in female mountain gorillas in the Virunga Volcanoes (Rwanda) and test four functional hypotheses, namely reconciliation, affiliation, dominance expression and sexual arousal. Homosexual interactions between females involved both ventro-dorsal and ventro-ventral copulations accompanied by vocalizations and courtship displays. The only sociosexual hypothesis that received partial empirical support is the social status hypothesis, i.e., that mounting reaffirms the dominance hierarchy. There is also some limited evidence that same-sex behavior reflects an overall state of arousal or is triggered via a ‘pornographic’ effect. An adaptive function of female homosexual behavior is not readily apparent, and we tentatively conclude (until a more rigorous test becomes available) that it may simply be related to sexual gratification or that it is an evolutionary by-product of an adaptation.

## Introduction

Same-sex or homosexual behavior–both between males and females–has been observed in many mammalian species [1, 2]. Given that it does not produce any reproductive benefits to the performers, it seems to be at odds with evolutionary theory and has puzzled evolutionary biologists for a long time. Various functional explanations have been offered (for a review see [3]); among the socio-sexual functions are dominance expression, social tension regulation, reconciliation, social bonding, alliance formation, acquisition of alloparental care, mate attraction, inhibition of competitor’s reproduction, practice for heterosexual activities, and kin selection. Others have also emphasized the non-functionality of this behavior, arguing that it might be a functionless by-product of evolution or a pathology (reviewed in [3], see also [4]).

From a phylogenetic perspective, tracing back the evolutionary occurrence of homosexual behavior might contribute to an understanding of such behavior in humans, which are unique among primates for not only engaging in homosexual acts, but also forming homosexual pair bonds [5]. Same-sex sexual behavior exists in all great apes: it is common and varied among bonobos [6, 7], but rare or absent among chimpanzees [8, 9], and orang-utans [10, 11]. As for gorillas, the only detailed accounts we have is on male-male interactions in mountain gorillas–mostly in bachelor groups [12–14]. There have also been reports of same-sex sexual interactions between immature individuals and between adults and immatures [2, 13, 15]. Only cursory evidence pertaining to female-female genital contact in gorillas has been presented, mostly from captivity [16, 17]. Fischer and Nadler [16] described infrequent sexual interactions between female western gorillas that included prolonged thrusting, typically in a ventro-ventral position. All that is known on female homosexuality from wild (mountain) gorillas was summarized in one paragraph by Harcourt et al. [18] and in a few anecdotal sentences by Fossey [19] (pp. 191–192). Fossey [19] noted that same-sex genital contact occurred in the later stages of pregnancy before labor and included copulatory vocalizations or pelvic thrusting and that such behavior reinforces the pregnant females’ social bonds within the group before parturition. Harcourt et al. [18] mentioned ten instances of homosexual mounts between females, both in ventro-ventral and dorso-ventral positions, including thrusts and usually with accompanying copulatory vocalizations from at least one of the partners. They interpreted this behavior as purely sexual in context, because mounts happened often during times of augmented sexual activity of females with males.

Here we provide the first formal report of homosexual behavior in female gorillas/mountain gorillas. We also attempt to test three of the most frequently invoked hypotheses regarding the functional significance of this pattern and an additional hypothesis. Given the somewhat limited number of observations and partial lack of critical information to assess competing hypotheses, this is considered a pilot endeavor. 1) The *reconciliation hypothesis* proposes that genital contacts are intended for resolving conflicts [7, 20]. This hypothesis has been examined in bonobos and Hanuman langurs, with mixed results. Hohmann and Fruth [21] found that genital contacts among female bonobos did not occur in conjunction with agonistic incidents, but rates rose during post-aggression phases. The work of Sommer et al. [22] did not accrue any findings in support of this theory. For the present study, evidence in favor of the reconciliation hypothesis would be shown if homosexual behavior is closely preceded by agonistic interactions. 2) According to the *social bond* hypothesis, homosexual behavior functions as a means of expressing or strengthening affiliative bonds [23]. Previous data were not or only partly in agreement with this hypothesis ([6, 21, 22], but see[24]). We predict a higher frequency of homosexual genital contacts among females with close ties, as measured by grooming, and/or among kin. 3) The *social status hypothesis* postulates that same-sex interactions represent ritualized dominance displays that help reaffirm the hierarchy, with the mounter thought to be dominant over the mountee [25–28]. Some primate studies have generated empirical support for this hypothesis [6, 21, 29], whereas others have not [30, 31]. If same sex contacts are related to dominance, we would predict high ranking individuals to be ‘on top’ more often than low ranking ones. 4) The *sexual arousal hypothesis* posits that homosexual behavior occurs more frequently at times when heterosexual activities are most common.

## Methods

Research was conducted on the habituated mountain gorilla population monitored by the Karisoke Research Center (KRC) of the Dian Fossey Gorilla Fund International (DFGFI) in Volcanoes National Park, Rwanda. The area consists of several physiognomically and taxonomically distinct vegetation assemblages, with Hagenia woodland interspersed with open herbaceous patches being the most frequently used ecotype by the gorillas [32].

In the Karisoke study area, mountain gorillas form either one-male or multi-male cohesive groups [33, 34], with groups ranging in size from 5 to 46 (KRC data for December 2010). The gorillas mainly feed on herbaceous plants [35, 36]. Mating happens primarily during the time of ovulation [37]. Both males and females emigrate [38]. In general, females are primarily attracted to protective males and develop weaker relationships with other females [39–41]. Dominance relationships among female mountain gorillas are often inconspicuous but detailed examinations of approach-retreat interactions suggest that a hierarchical order is prevalent and stable over the long term [42, 43]. Grooming between females is uncommon, but is more common among relatives [44]. Reconciliation after conflicts is rare between females [45].

Data on same-sex sexual behavior were obtained from two research groups (Pablo, Bwenge). All individuals in the study groups could be individually recognized. Group Pablo contained 46 and Bwenge 10 individuals (data as of December 2010). These two groups were observed on a nearly daily basis from January 2008 to December 2010 for a maximum of four hours per day. All observations were conducted in compliance with regulations of the Rwanda Development Board–Tourism and Conservation which dictate that at all times a minimum distance of 7 m needs to be kept between the gorillas and human observers. Data collection involved focal sessions lasting 50 min during which all occurrences of social (e.g. allogrooming) and sexual behavior (including solicitation), dominance-related (displacements) and aggressive behavior (both light threats and heavy aggression such as contact aggression) involving the focal animal were recorded. Homosexual behavior, displacements and intense aggression were also recorded ad lib outside of focal sessions. All subadult females, nulliparous adult females and adult females were included in the study. Additional observations on homosexuality were made using all-occurrence sampling as part of a study on feeding ecology which included 30-min focal sessions of females. In an attempt to garner additional descriptive evidence of homosexual behavior in female gorillas, questionnaires were also distributed to all data technicians working at KRC.

Homosexual mounting was defined as an individual climbing on top of another individual, ventro-dorsally or ventro-ventrally, with or without pelvic thrusting; it does not include mounting during play (distinguished by play face, play grunts) and it also does not include brief embraces. Mounts involving the same dyad had to be separated by at least 15 min to count as separate events. Mounts were not only observed between females, but also between other age-sex classes (blackbacks and infants, subadult females and infants, blackbacks and subadult males), but these were not included in the present analysis.

Dominance relationships were assessed via displacement events which were put into a matrix and analyzed using the I&SI method in MATMAN (Noldus Information Technology) which ranks individuals into a linear hierarchy. Ordinal rankings were then standardized following [42] so that the lowest ranked female had a standardized rank of 0 and the highest ranking one a standardized rank of 1 (S1 Table). Rank changes were minimal across years. The strength of social bonds was assessed via grooming bouts involving particular dyads: for the purpose of this analysis, all female-female dyads that had grooming bouts during the observation period were considered ‘bonded’ whereas dyads with no record of grooming behavior were considered ‘non-bonded’ (S2 Table). The possible conciliatory function of homosexual behavior was assessed by extracting data on aggressive interactions involving a given dyad that occurred during a 10-minute time window preceding the homosexual event. 10 minutes was chosen since research on numerous primates has shown that most conflicts are usually resolved within the first minute of the post-conflict period [46]. The sexual arousal hypothesis was explored by recording if a female engaging in a homosexual act was also involved in a heterosexual copulation on the same day, previous day or following day.

Some of the circumstances surrounding homosexual acts were not systematically recorded (such as response of silverback, event preceding mounts etc.), but were reconstructed based on interviews with data technicians and in some cases detailed observations by the lead author. Qualitative evidence is presented alongside systematically collected data wherever available.

The occurrence of seasonal clumping of mounts was analyzed using a Kolmogorov-Smirnov test. The association between copulation events and grooming events was analyzed using a Spearman rank correlation. A chi square test was used to compare the frequencies with which dominants vs. subordinates occupied the top position during mounting. All p-values reported are two-tailed.

This research adheres to the laws of the country where the research was conducted. All applicable international, national, and/or institutional guidelines for research on animals were followed. All procedures performed were in accordance with the ethical standards of the institutions at which this research was conducted. Research permits were issued by Rwanda Development Board.

## Results

We recorded a total of 44 sexual contacts between females. 14 (of an average of 16) females in group Pablo and 4 (of an average of 6) females in group Bwenge engaged in homosexual behavior. Of all cases observed, 30 involved two adult females, four involved two subadult females and ten involved one adult and one subadult female. Some individuals exhibited a higher propensity for same-sex acts, e.g. AFR and IEA in group Pablo were seen engaging in homosexual behavior 11 and 8 times, and MAG and NZE in group Bwenge were seen 12 and 9 times, respectively (S2 Table). These numbers are only slightly lower than those of male-female copulations over the same time period: AFR: 22 times, IEA: 13 times, MAG: 19 times, and NZE: 16 times (KRC, unpublished data). A total of 26 different dyads were observed participating in homosexual acts (S1 Table). Some dyads were also more likely to exhibit homosexual behavior, esp. MAG-NZE with 7 recorded instances. Females of all reproductive states were found to show homosexual behavior; the breakdown for dyads according to reproductive state is: 5 cycling-cycling, 6 cycling-lactating, 21 cycling-pregnant, 1 pregnant-lactating, 8 pregnant-pregnant, and 2 lactating-lactating. There was no seasonal clumping of mounts, with the monthly distribution of events not being different from a uniform distribution (Kolmogorov-Smirnov test, Z = 0.751, p = 0.626, n = 12 months).

**Fig 1:**
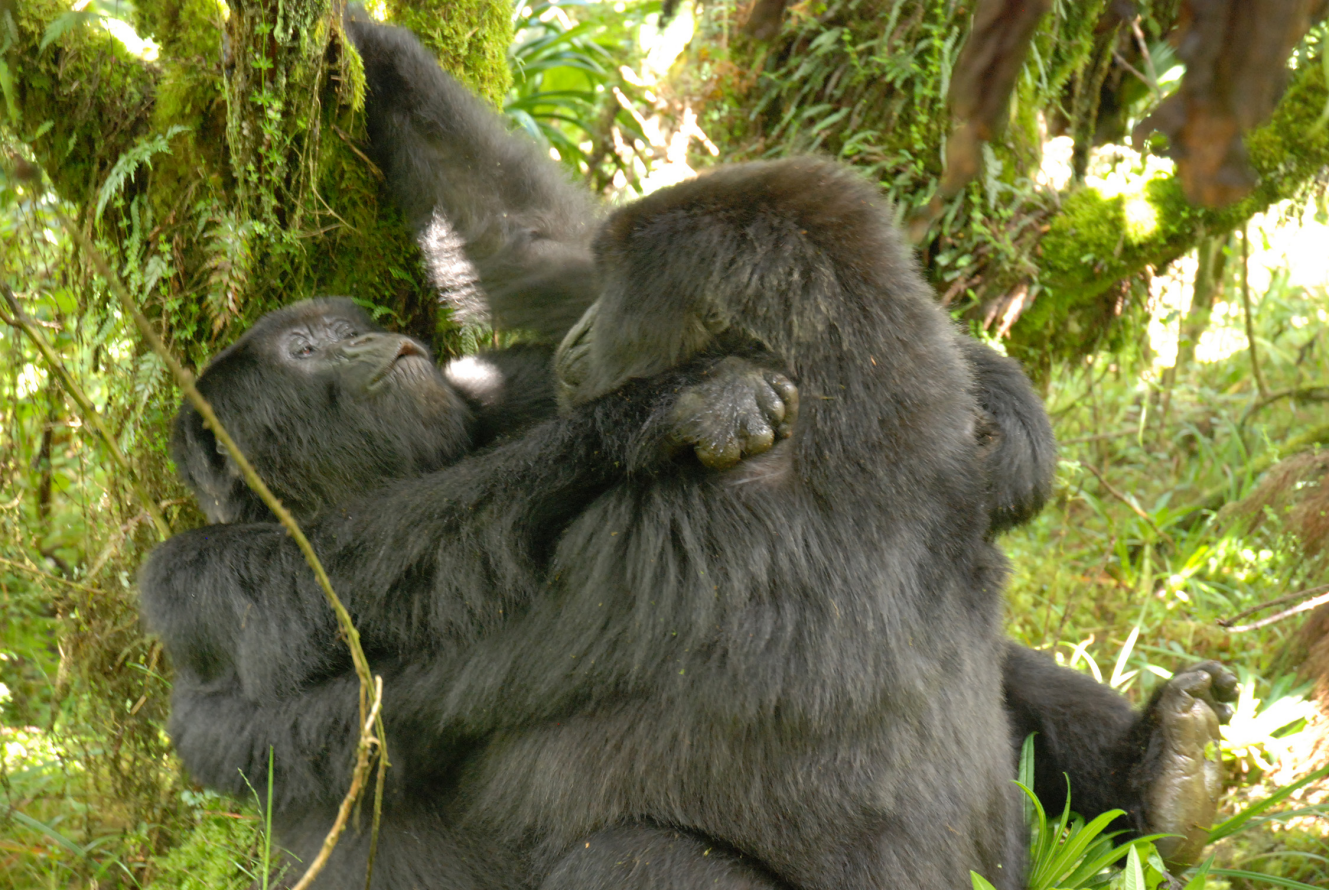
Two females in group Pablo engaging in ventro-ventral homosexual mounting (frottage). The mountee is uttering copulatory vocalizations. Photo: Cyril C. Grueter.

Same-sex copulations happened both ventro-ventrally and ventro-dorsally (Fig 1). Copulations for which duration was recorded (n = 6) lasted on average 85 seconds (range: 60–180 seconds). Copulations were typically accompanied by calls given in ‘real’ heterosexual copulations, i.e. the typical trill call. Most copulations involved solicitation behavior or courtship displays whereby a female would cautiously approach the partner and then stand facing partner with her body slightly turned to the side (as seen in heterosexual solicitation, [17]). There was also a tendency for such copulations to take place in secluded places with dense vegetation. Silverbacks sometimes reacted aggressively toward the copulating pair. The dominant silverback BWE showed aggressive behavior to the copulating pair MAG-NZE in five cases (three times mild, twice physical). On 05 May, 2010, BWE bit NZE after she had mounted FAI. In one case when INT was copulating with IEA, dominant silverback CAN pig-grunted at INT. In another case in group Pablo (May 20, 2010), the copulating pair (NYB-AFR) received mild threat calls by silverback GIC, second-in command. Aggressive interruptions of sexual acts by silverbacks were also seen by data technicians observing other groups. However, the witness of a homosexual mount by a silverback did not always result in aggressive intervention, e.g. when UMC and MAH engaged in a copulatory act on Jul 26, 2010, the dominant silverback CAN was resting right next to them and did not interfere.

### Homosexual behavior and reconciliation

None of the homosexual acts were preceded by aggressive events in the 10 min before, but some mild threats such as ‘pig grunt’ calls may well have been missed as they were not consistently recorded. In one case when pig grunts were recorded between UMC and MAH (July 26 2010), a homosexual act followed within less than 1 min after the aggressive event.

### Homosexual behavior and social bonds

None of the homosexual acts involved close kin dyads (mother-daughter, maternal sisters), but one was aunt-niece (AFR-MAH). To establish if homosexual behavior is more common among females that affiliate with each other, we compared the frequency of acts involving non-bonded individuals (n = 21) with those involving bonded individuals (n = 22) and found no statistical difference. In group Bwenge, almost all dyads that copulated also affiliated, so we correlated number of copulation events with number of grooming events, but there was no relationship (r_s_ = 0.112, p = 0.858, n = 5).

### Homosexual behavior and dominance

**Fig 2:**
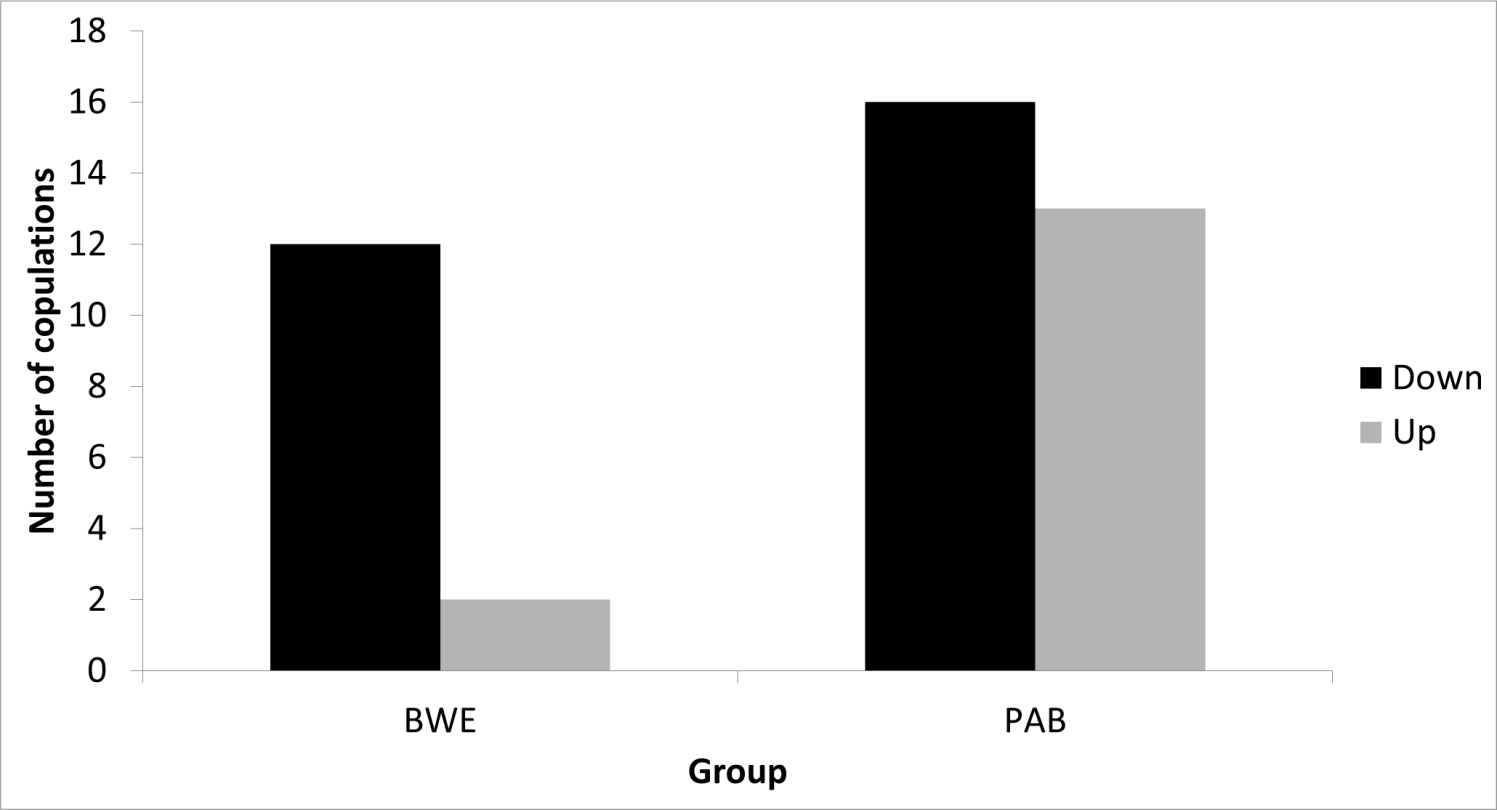
Relative dominance ranks of mounter and mountee (‘mounting direction’). ‘Down’ = down the hierarchy, ‘up’ = up the hierarchy.

A total of 43 mounts were observed for which the identities of both participants were known, 29 in group Pablo, and 14 in group Bwenge. For both groups combined, there was a barely significant difference in the frequencies of mounting direction (chi square = 3.93, p = 0.0474). In group Pablo, significance was lost completely (chi square = 0.31, p = 0.577). In group Bwenge, most copulations were ‘top-down’, with the mounter being higher-ranked than the mountee (chi square = 7.143, p = 0.0075) (Fig 2); this result was clearly driven by one individual (MAG).

### Homosexual behavior and sexual arousal

Twelve out of 43 homosexual events (28%) involved at least one female that was also involved in a heterosexual act on the same/preceding/following day.

## Discussion

Our report demonstrates that same-sex sexual contacts among females are clearly a component of the behavioral repertoire of mountain gorillas, albeit a relatively infrequent one. Given that such homosexual interactions happened in a completely wild setting, the claim that it is an artifact of captivity is not applicable. Genital contacts between females have also been documented in some other mountain gorilla groups in the Karisoke study area (e.g. former Shinda’s group, KRC), so this behavior does not seem to be a group-specific tradition. Since female homosexual behavior has also been observed in the Bwindi (Uganda) population of mountain gorillas (Robbins, unpublished data), it also does not constitute a population-specific tradition (as has been argued for genital rubbing in female orangutans [47].

Heterosexual copulations are usually dorso-ventral ([15, 18]; but see [48], Grueter, personal observations). The reason why some genital contacts between females were ventro-ventral (similar to bonobos) is probably because this position ensures maximal sexual stimulation (vulvae in contact). Ventro-ventral mounting has also been described for male mountain gorillas [12].

Males were sometimes observed to attack females, which was also reported by [18]. While sexual interference in heterosexual episodes has been interpreted as an adaptive strategy to reduce paternity uncertainty [49], interference in homosexual acts could be regarded as an evolutionary by-product of such an adaptation. A reason why copulations tended to take place in secluded places with limited visibility is probably to avoid a negative response by the silverback.

Finding a suitable evolutionary explanation for homosexual behavior in any primate is challenging. In the case of the mountain gorillas studied here, some hypotheses can be discarded *a priori*, e.g. that mounting is aimed at training and practice (cf. [50], since mounts often involved highly ‘experienced’ females and subadult females were not overrepresented.

The *social status hypothesis*, i.e. that directionality of mounting is an indicator of social rank, was only partly supported by the data (fully supported in group Bwenge, but not in Pablo). Overall, the result in group Bwenge are heavily influenced by one idiosyncratic individual, viz. MAG that was responsible for most of the top position mounts. Data on initiation of genital contacts were not systematically collected in this study. In bonobos, dominant females occupied the top position significantly more often than the bottom position [6, 7]. A recent study by Clay and Zuberbühler [31] found that high-ranking females did not occupy the top position more often than low-ranking females, but genital contacts were more likely to be initiated by dominant females. In Hanuman langurs, female mounters of high rank were significantly overrepresented in comparison to middle- and low-ranking classes, but among mountees, low-ranking individuals were not overrepresented [22]. Vasey’s [51] extensive data set on female Japanese macaques did not produce any support for the prediction that positions individuals take during same-sex mounting are discriminated on the basis of rank. One problem with the dominance expression hypothesis is that it ascribes a submissive role to the mountee and dominant role to the mounter, a concept that may be open to interpretation [22, 52], particularly given that many copulations were ventro-ventral. Moreover, the observation that at least in some cases affiliative and courtship behavior preceded and initiated copulations would not be expected if mounts function to reaffirm the dominance hierarchy.

The *social bonding hypothesis* can also be tentatively rejected based on the preliminary evidence obtained so far. Most mounts involved individuals that did not seem to exhibit strong social bonds or affiliative ties, as measured by grooming (same as in bonobos [6]). Sommer et al. [22] found a negative correlation between ratio of grooming/displacement (a measure of relationship quality) with dyadic rates of same-sex mounting in Hanuman langurs, indicating that mounting is not a sign of a strong social relationship. In bonobos, same-sex mounting between newly immigrant young females and between young females and high-ranking old adult females was frequently recorded, but grooming was uncommon in such dyads [24]. In the case of mountain gorilla females, it could be that social grooming between close affiliates is sufficient for relationship maintenance (with the added benefit of hygiene [22]. We found no effect of kinship, a finding that is consistent with what has been shown for Japanese macaques where sexual behavior was never observed between close female kin [53].

Mounting can be a conciliatory gesture in some primates [54, 55]. Our data are not in favor of the *reconciliation hypothesis*, as the bulk of mounts were not preceded by agonistic encounters. For bonobos, the reconciliation function was highlighted in one captive study [7], but this may be an artifact of captivity. In a detailed wild study rates of genital contacts increased after agonistic interactions, thus offering some support to the reconciliation hypothesis, but a substantial proportion of homosexual behavior could not be explained by agonism [6]. Hohmann and Fruth [21] argue that lack of immediate expression of genital contacts after conflict does not indicate lack of support for the reconciliation hypothesis, as the crucial variable here might be an increase in rates of such contacts following aggression. In Japanese macaques, same-sex sexual behavior did not occur more frequently after conflicts [51]. In Hanuman langurs, only 7.5% of same-sex mounts between females occurred in the context of conflict between group members [22]. Considering that female bonds [44], dominance relationships ([56], but see [42]) and reconciliation [45] are all relatively poorly expressed in mountain gorillas, it comes as no surprise that these do not seem to explain homosexual acts between females.

We did not evaluate the hypothesis that homosexual mounts are a means by which tension over access to limited food is regulated *a priori* (as opposed to post-conflict reunions). This hypothesis has received mixed support in bonobos [6, 57]. Given the rather even distribution of food in most parts of the gorillas’ habitat and lack of usurpability of resources (but see [43]), it is less likely that sex plays a role in feeding competition.

In our study 72% of the homosexual mounts took place without any obvious relation to heterosexual copulations although accurate quantification was impeded by the possibility that some copulations were missed. There were some cases of mounters, mountees or both engaging in a heterosexual copulation during a 48-hour window surrounding the homosexual bout. Harcourt et al. [18] recorded six occasions of females copulating with the male on the day of the homosexual mount, and further noted that in five of these six occasions it was the estrous female that thrusted, the other acting passively. The majority of incidents of homosexual behavior in our study (10/14) involved pregnant and lactating females, i.e. they were not at a point where they could have been ovulating. A concurrence with ‘estrous’ periods is therefore an unlikely explanation for performing a homosexual act, but general sexual arousal may still play a role in triggering homosexual behavior. In one case, AFR mounted IEA and just 3 min later AFR mounted GUT which could be an indication of sexual arousal. On another day, MUK mounted UMC and 37 min later she mounted NYB (the next day silverback CAN copulated with MUK). In Hanuman langurs, the frequency of same-sex mounting was higher than expected during the periovulatory phase [22]. In bonobos, Fruth and Hohmann [6] found no clear evidence that female homosexual behavior incited heterosexual copulation activities. A recent study reports that frequencies of genito-genital rubbing increased in maximally tumescent bonobo females, suggesting a link between estrus state and homosexual behavior [58].

A variant of the above explanation (sexual arousal) is the sexual dissatisfaction or redirected copulation hypothesis according to which a female seeks a same-sex replacement partner when a male ignores her advances [59, 60]. We have seen such cases, but for a systematic assessment we would need data on male presence and behavior. In proboscis monkeys, female-female mounts were recorded shortly after failed solicitations toward males [61], and female-female mounts could therefore be attributed to ‘sexual frustration’. In our study, in at least four of the above described cases (e.g. May 20 and 21, 2010) females sometimes also solicited to a silverback that did not show any interest, and females then turned to other females. Females soliciting copulations from a silverback prior to same-sex mounts was also observed by KRC data technicians working in other groups.

While enhanced opposite-sex sexual arousal may elevate the possibility of same-sex behavior, such behavior may also be activated at other times via a ‘pornographic effect’, i.e. a female witnessing sexual behavior and getting aroused. In order to test this, data are needed on whether copulations between any two individuals precede homosexual behavior. We have recorded a few cases in which a short time (less than one minute to twelve minutes) after an adult male initiated a copulation with a female, a bystander female would solicit and carry out a copulation with yet another uninvolved female: In three cases of MAG-NZE copulations, there was a copulation between silverback BWE and KWR shortly before (once twelve minutes before, twice three minutes before). In none of the cases did MAG solicit to BWE; she actually solicited to NZE. In another case, BWE copulated with NTO, and 11 min later MAG copulated with KWR. There was another case when MAG started copulating with an unidentified female right after BWE initiated a copulation with KWR.

It has also been proposed that females use mounts as an instrument to attract the male’s sexual interest [62]. A formal test of this hypothesis would require data on whether a male was nearby or out of sight during such events.

Yet another untested ultimate hypothesis is the female competition hypothesis, i.e. that a female mounter uses homosexuality as a strategy to decrease a mountee’s motivation to copulate, which can negatively impact a same-sex competitor’s reproductive success [52]. Under this scenario, females should then preferably do this when the mountee is sexually receptive. A similar expression of reproductive female-female competition has been documented for western gorillas in both the wild and a zoo environment who exhibit post-conceptive mating, presumably to minimize male interest in other females and in so doing delay conception in others [63, 64].

While work on some primates has revealed sociosexual functions of homosexual behavior (see above), some investigators have concluded that homosexual behavior in primates may be purely sexual [6, 22, 51]. The functional explanations seem to be a rather poor fit to the observed pattern of homosexual behavior between female mountain gorillas although there was partial support for the hypothesis that directionality of mounting is an indicator of social rank. Homosexual behavior in *male* mountain gorillas appears to have no socio-sexual function [12], e.g. it was not an expression of dominance since mounters were not generally higher ranking [13]. It is conceivable that sexual pleasure/satisfaction is the main benefit females derive from engaging in same-sex mounting (see also [18]). The fact that homosexual acts involved genital closeness and were accompanied by copulation calls is an indication that sexual gratification occurred. Moreover, unrelated females rarely engage in close socio-positive contacts such as grooming or cuddling, so homosexual behavior is different in its manifestation and intensity from same-sex *social* interactions; it is thus parsimonious to assume that same-sex genital contact is sexually/hedonically motivated.

Vasey [50, 51, 65] proposed a by-product theory for the evolution of homosexuality in female primates, arguing that homosexual behavior is not counter-selected since it does not inhibit reproduction of the partakers. He hypothesized that homosexual behavior in Japanese macaques is not adaptive, but evolved as a by-product of selection for *female*-*male* mounting which females use strategically to encourage sexually disinterested males to copulate with them. This generates sexual pleasure through clitoral stimulation. This reward could have also been obtained from female-female mounts, which then became fixed in the population. This explanation does not apply to gorillas, however, since they do not show such behavior.

From a comparative angle, it is evident that socio-sexual interactions among female mountain gorillas are not nearly as prominent as in bonobos which likely reflects weaker female bonding in the former as compared to the latter. For bonobos it has been postulated that same-sex sexual contacts strengthen female relationships and allow them to establish a ‘second sisterhood’ as a counterstrategy against male coercion ([24], see also [66]). In line with their predominantly male-bonded societies, female homosexual behavior is rare in chimpanzees.

In sum, while there was limited support for the social status hypothesis, homosexual behavior in female mountain gorillas may well be of purely sexual nature. More systematic studies collecting additional information and testing alternative hypothesis will help illuminate a possible adaptive function.

## Supporting Information

S1 Table Frequency of homosexual bouts and dominance rank for all females. (DOCX)

S2 Table Contextual details on female-female homosexual events. (DOCX)

## References

[1] Bagemihl B. Biological Exuberance: Animal Homosexuality and Natural Diversity. New York: St Martin's; 1999.

[2] Furuichi T, Connor R, Hashimoto C. Non-conceptive sexual interactions in monkeys, apes, and dolphins In: Yamagiwa J, Karczmarski L., editors. Primates and Cetaceans: Field Research and Conservation of Complex Mammalian Societies. Tokyo: Springer p. 385–408.

[3] Vasey PL, Sommer V. Homosexual behaviour in animals: topics, hypotheses and research trajectories In: Sommer V, Vasey PL, editors. Homosexual Behaviour in Animals: An Evolutionary Perspective. Cambridge: Cambridge University Press; 2006 p. 3–42.

[4] Bailey NW, Zuk M. Same-sex sexual behavior and evolution. TRENDS IN ECOLOGY AND EVOLUTION. 2009;24:439–46. 19539396 10.1016/j.tree.2009.03.014

[5] Kirkpatrick RC. The evolution of human homosexual behavior. Current Anthropology. 2000;41:385–413. 10768881

[6] Fruth B, Hohmann G. Social grease for females? Same-sex genital contacts in wild bonobos In: Sommer V, Vasey PL, editors. Homosexual Behaviour in Animals: An Evolutionary Perspective. Cambridge: Cambridge University Press; 2006 p. 294–315.

[7] De Waal FBM. Tension regulation and nonreproductive functions of sex in captive bonobos (*Pan paniscus*). Nat Geogr Res. 1987;3:318–38.

[8] Goodall J. In the Shadow of Man. Revised ed. Boston: Houghton Mifflin; 1988.

[9] Anestis SF. Female genito-genital rubbing in a group of captive chimpanzees. International Journal of Primatology. 2004;25:477–88.

[10] van Schaik CP. Orangutan cultures and the comparative study of culture. American Journal of Physical Anthropology. 2003:214–.

[11] Fox EA. Homosexual behavior in wild Sumatran orangutans (*Pongo pygmaeus abelii*). American Journal of Primatology. 2001;55:177–81. 11746281 10.1002/ajp.1051

[12] Yamagiwa J. Playful encounters: the development of homosexual behaviour in male mountain gorillas In: Sommer V, Vasey PL, editors. Homosexual Behaviour in Animals: An Evolutionary Perspective. Cambridge: Cambridge University Press; 2006 p. 273–93.

[13] Yamagiwa J. Intra- and inter-group interactions of an all-male group of Virunga mountain gorillas (*Gorilla gorilla beringei*). Primates. 1987;28:1–30.

[14] Robbins MM. Male-male interactions in heterosexual and all-male wild mountain gorilla groups. Ethology. 1996;102:942–65

[15] Harcourt AH, Fossey D, Stewart KJ, Watts DP. Reproduction in wild gorillas and some comparisons with chimpanzees. J Reprod Fert. 1980;Suppl 28:59–70. 6934312

[16] Fischer RB, Nadler RD. Affiliative, playful, and homosexual interactions of adult female lowland gorillas. Primates. 1978;19:657–64.

[17] Hess JP. Some observations on the sexual behavior of captive lowland gorillas, *Gorilla g*. *gorilla* (Savage and Wyman) In: Michael RP, Crook JH, editors. Comparative Ecology and Behavior of Primates. London: Academic Press; 1973 p. 507–81.

[18] Harcourt AH, Stewart KJ, Fossey D. Gorilla reproduction in the wild In: Graham C, editor. Reproductive Biology of the Great Apes. New York: Academic Press; 1981 p. 265–279.

[19] Fossey D. Gorillas in the Mist. Boston: Houghton Mifflin; 1983.

[20] Dixson AF. Observations on the displays, menstrual cycles and sexual behavior of the 'Black ape' of Celebes (*Macaca nigra*). Journal of Zoology. 1977;182:63–84.

[21] Hohmann G, Fruth B. Use and function of genital contacts among female bonobos. Animal Behaviour. 2000;60:107–20. 10924210 10.1006/anbe.2000.1451

[22] Sommer V, Schauer P, Kyriazis D. A wild mixture of motivations: same-sex mounting in Indian langur monkeys In: Sommer V, Vasey PL, editors. Homosexual Behaviour in Animals: An Evolutionary Perspective. Cambridge: Cambridge University Press; 2006.

[23] Parish AR. Female relationships in bonobos (*Pan paniscus*): evidence for bonding, cooperation, and female dominance in a male-philopatric species. Human Nature. 1996;7:61–96. 10.1007/BF02733490 24203252

[24] Furuichi T. Social interactions and the life history of female *Pan paniscus* in Wamba, Zaire. International Journal of Primatology. 1989;10:173–197.

[25] Dagg AI. Homosexual behavior and female-male mounting in mammals—a first survey. Mammal Rev. 1984;14:155–85.

[26] Chadwick-Jones JK. Presenting and mounting in non-human primates: theoretical developments. J Soc Biol Struct. 1989;12:319–33.

[27] Nadler RD. Homosexual behavior in nonhuman primates In: McWhirter DP, Sanders SA, Reinisch JM, editors. Homosexuality/Heterosexuality: Consepts of Sexual Orientation. New York: Oxford University Press; 1990 p. 138–70.

[28] Akers JS, Conaway CH. Female Homosexual Behavior in *Macaca mulatta* Archives of Sexual Behavior. 1979;8:63–80. 105688 10.1007/BF01541214

[29] Talmage-Riggs G, Anschel S. Homosexual behavior and dominance hierarchy in a group of captive female squirrel monkeys (*Saimiri sciureus*). Folia Primatologica. 1973;19:61–72. 10.1159/000155519 4198058

[30] Vasey PL, Chapais B, Gauthier C. Mounting interactions between female Japanese macaques: testing the influence of dominance and aggression. Ethology. 1998;104:387–98.

[31] Clay Z, Zuberbühler K. Communication during sex among female bonobos: effects of dominance, solicitation and audience. Scientific Reports. 2012;2:Article number 291. 10.1038/srep00291 PMC3291041 22389761

[32] McNeilage A. Diet and habitat use of two mountain gorilla groups in contrasting habitats in the Virungas In: Robbins MM, Sicotte P, Stewart KJ, editors. Mountain Gorillas: Three Decades of Research at Karisoke. Cambridge: Cambridge University Press; 2001 p. 265–92.

[33] Robbins MM. Variation in the social system of mountain gorillas: The male perspective In: MM R, P S, KJ S, editors. Mountain Gorillas: Three Decades of Research at Karisoke. New York: Cambridge Univ Press; 2001 p. 29–58.

[34] Stewart KJ, Harcourt AH. Gorillas: variation in female relationships In: Smuts BB, Cheney DL, Seyfarth RM, Wrangham RW, Struhsaker TT, editors. Primate Societies. Chicago: University of Chicago Press; 1987 p. 155–64.

[35] Watts DP. Composition and variability of mounain gorilla diets in the Central Virungas. American Journal of Primatology. 1984;7:323–56. 10.1002/ajp.1350070403 32106635

[36] Grueter CC, Ndamiyabo F, Plumptre AJ, Abavadimwe D, Fawcett KA, Robbins MM. Long-term temporal and spatial dynamics of food availability for endangered mountain gorillas in Volcanoes National Park, Rwanda. American Journal of Primatology. 2013;75:267–80. 10.1002/ajp.22102 23208819

[37] Czekala NM, Sicotte P. Reproductive monitoring of free-ranging female mountain gorillas by urinary hormone analysis. American Journal of Primatology. 2000;51:209–15. 10902670 10.1002/1098-2345(200007)51:3<209::AID-AJP6>3.0.CO;2-6

[38] Harcourt AH, Stewart KJ, Fossey D. Male emigration and female transfer in wild mountain gorillas. Nature. 1976;263:226–7.

[39] Harcourt AH. Social relationships among adult female mountain gorillas. Animal Behaviour. 1979;27:251–64.

[40] Harcourt AH. Social relationships between adult male and female mountain gorillas. Animal Behavior. 1979;27:325–42.

[41] Watts D. Gorilla social relationships: A comparative overview In: AB T, ML G, editors. GORILLA BIOLOGY: A MULTIDISCIPLINARY PERSPECTIVE. New York: Cambridge Univ Press; 2003 p. 302–27.

[42] Robbins MM, Robbins AM, Gerald-Steklis N, Steklis HD. Long-term dominance relationships in female mountain gorillas: strength, stability and determinants of rank. Behaviour. 2005;142:779–809.

[43] Grueter CC, Robbins AM, Abavadimwe D, Vecellio V, Ndagijimana F, Ortmann S, et al Causes, mechanisms, and consequences of contest competition among female mountain gorillas in Rwanda. in review

[44] Watts DP. Social relationships of immigrant and resident female mountain gorillas, II: Relatedness, residence, and relationships between females. American Journal of Primatology. 1994;32:13–30. 10.1002/ajp.1350320103 31936904

[45] Watts DP. Post-conflict social events in wild mountain gorillas, 1. Social interactions between opponents. Ethology. 1995;100:158–74.

[46] Arnold K, Fraser ON, Aureli F. Postconflict reconciliation In: Campbell CJ, Fuentes A, MacKinnon KC, Bearder SK, Stumpf RM. Editors. Primates in Perspective. 2nd edition New York: Oxford University Press; 2011 p. 608–625.

[47] van Schaik CP, Ancrenaz M, Borgen G, Galdikas B, Knott CD, Singleton I, et al Orangutan cultures and the evolution of material culture. Science. 2003;299:102–5. 12511649 10.1126/science.1078004

[48] Breuer T, Ndoundou Hockemba M. Ventro-ventral copulation in gorillas. Gorilla Gazette. 2007;20:47–9.

[49] Neimeyer CL, Anderson JR. Primate harassment of matings. Ethology and Sociobiology. 1983;4:205–20.

[50] Vasey PL. Homosexual behavior in primates: a review of evidence and theory. International Journal of Primatology. 1995;16:173–204.

[51] Vasey PL. The pursuit of pleasure: an evolutionary history of female homosexual behaviour in Japanese macaques In: Sommer V, Vasey PL, editors. Homosexual Behaviour in Animals: An Evolutionary Perspective. Cambridge: Cambridge University Press; 2006 p. 191–219.

[52] Srivastava A, Borries C, Sommer V. Homosexual mounting in free-ranging female Hanuman langurs (*Presbytis entellus*). Arch Sex Behav. 1991;20:487–512. 1747043 10.1007/BF01542410

[53] Chapais B, Gauthier C, Prud'homme J, Vasey PL. Relatedness threshold for nepotism in Japanese macaques. Animal Behaviour. 1997;53:533–48.

[54] de Waal FBM, van Roosmalen A. Reconciliation and consolation among chimpanzees. Behavioral Ecology and Sociobiology. 1979;5:55–66.

[55] Grueter CC. Conflict and postconflict behavior in captive black-and-white snub-nosed monkeys (*Rhinopithecus bieti*) Primates. 2004;45:197–200. 10.1007/s10329-004-0077-9 15042414

[56] Watts DP. Agonistic relationships between female mountain gorillas (*Gorilla gorilla beringei*). Behavioral Ecology and Sociobiology. 1994;34:347–58.

[57] White FJ, Lanjouw A. Feeding competition in Lomako bonobos: variation in social cohesion In: Nishida T, McGraw WC, Marler P, Pickford M, de Waal FBM, editors. Topics in Primatology, Vol 1, Human Origins Tokyo: University of Tokyo Press; 1992 p. 67–79.

[58] Ryu H., Hill DA, Furuichi T. Prolonged maximal sexual swelling in wild bonobos facilitates affiliative interactions between females. Behaviour 2015;152:285–311.

[59] Hanby JP. Male-male mounting in Japanese monkeys (*Macaca fuscata*). Animal Behaviour. 1974;22:152–95. 10.1016/0003-3472(74)90006-2 4462407

[60] Poiani A. Animal Homosexuality: A Biosocial Perspective Cambridge Cambridge University Press; 2010.

[61] Murai T. Mating behaviors of the proboscis monkey (*Nasalis larvatus*). American Journal of Primatology. 2006;68:832–7. 16847976 10.1002/ajp.20266

[62] Parker GA, Pearson RG. A possible origin and adaptive significance of the mounting behavior shown by some female mammals in oestrous. J Nat Hist. 1976;10:241–5.

[63] Stoinski TS, Perdue BM, Legg AM. Sexual behavior in female western lowland gorillas (*Gorilla gorilla gorilla*): evidence for sexual competition. American Journal of Primatology. 2009;71:587–93. 10.1002/ajp.20692 19399838

[64] Doran-Sheehy DM, Fernández D, Borries C. The strategic use of sex in wild female western gorillas. American Journal of Primatology. 2009;71:1011–20. 10.1002/ajp.20743 19722225

[65] Vasey PL. Same-sex sexual partner preference in hormonally and neurologically unmanipulated animals. Ann Rev Sex Res. 2002;13:141–79. 12836731

[66] Furuichi T. Female contributions to the peaceful nature of bonobo society. Evolutionary Anthropology 2011;20:131–142. 10.1002/evan.20308 22038769

