# Supplementary material for: Homosexual Behavior in Female Mountain Gorillas: Reflection of Dominance, Affiliation, Reconciliation or Arousal?
Source: PLoS One. 2016 May 11;11(5):e0154185. doi: 10.1371/journal.pone.0154185 (PMC4864209; doi:10.1371/journal.pone.0154185)
Supplement: S1 Table — (DOCX) [file pone.0154185.s001.docx]

**S1 Table. Frequency of homosexual bouts and dominance rank for all females.**

|  | **No. of incidents of homosexual contact** | **Standardized rank** |
| --- | --- | --- |
| *Group PAB* | | |
| MAH | 6 | 0.94 |
| UMC | 3 | 1 |
| MUK | 5 | 0.71 |
| NYB | 5 | 0.77 |
| BKR | 4 | 0.47 |
| AFR | 11 | 0.53 |
| IEA | 8 | 0.06 |
| GUT | 7 | 0.65 |
| MIT | 1 | 0.41 |
| INT | 3 | 0.88 |
| MUD | 1 | 0.82 |
| TMS | 2 | 0.29 |
| ISU | 1 | 0.18 |
| NDW | 1 | 0 |
| *Group BWE* | | |
| MAG | 12 | 1 |
| KWR | 4 | 0.83 |
| NZE | 9 | 0.5 |
| FAI | 4 | 0 |
