# Supplementary material for: Homosexual Behavior in Female Mountain Gorillas: Reflection of Dominance, Affiliation, Reconciliation or Arousal?
Source: PLoS One. 2016 May 11;11(5):e0154185. doi: 10.1371/journal.pone.0154185 (PMC4864209; doi:10.1371/journal.pone.0154185)
Supplement: S2 Table — (DOCX) [file pone.0154185.s002.docx]

**S2 Table. Contextual details on female-female homosexual events.**

| **Date** | **Group** | **Mounter** | **Mountee** | **Age class mounter** | **Age class mountee** | **Mounting direction with regard to hierarchy** | **Aggression preceding homosexuality (10 min)** | **Bond (yes-no) [# grooming bouts per dyad in brackets]** | **Kin (mother-daughter or sisters)** | **Mounted with silverback on same day or +- 1 day (mounter)** | **Mounted with silverback on same day or +- 1 day (mountee)** | **Reprod. state mounter** | **Reprod. state mountee** |
| --- | --- | --- | --- | --- | --- | --- | --- | --- | --- | --- | --- | --- | --- |
| 14-Feb-08 | PAB | IEA | MUD | SAF | AF | Up | No | No | No | No | No | Cycling | Lactating |
| 20-Feb-08 | PAB | GUT | AFR | AF | SAF | Down | No | No | No | Yes | Yes | Pregnant | Cycling |
| 26-Feb-08 | PAB | IEA | AFR | SAF | SAF | Up | No | No | No | No | No | Cycling | Cycling |
| 2-Mar-08 | PAB | AFR | IEA | SAF | SAF | Down | No | No | No | No | No | Cycling | Cycling |
| 2-Mar-08 | PAB | AFR | GUT | SAF | AF | Up | No | No | No | No | No | Cycling | Pregnant |
| 8-Mar-08 | PAB | MIT | IEA | AF | SAF | Down | No | No | No | No | No | Lactating | Cycling |
| 27-Mar-08 | PAB | AFR | IEA | SAF | SAF | Down | No | No | No | No | No | Cycling | Pregnant |
| 4-Apr-08 | PAB | INT | IEA | AF | SAF | Down | No | No | No | No | No | Pregnant | Pregnant |
| 5-Apr-08 | PAB | GUT | INT | AF | AF | Up | No | No | No | No | No | Pregnant | Pregnant |
| 2-Jul-08 | PAB | INT | IEA | AF | SAF | Down | No | No | No | No | No | Pregnant | Pregnant |
| 26-Apr-09 | PAB | MUK | UMC | AF | AF | Up | No | No | No | Yes | Yes | Pregnant | Lactating |
| 26-Apr-09 | PAB | MUK | NYB | AF | AF | Up | No | Yes [3] | No | Yes | No | Pregnant | Pregnant |
| 27-Apr-09 | PAB | MAH | UMC | AF | AF | Up | No | No | No | No | Yes | Cycling | Lactating |
| 28-Apr-09 | PAB | MAH | NYB | AF | AF | Down | No | No | No | No | Yes | Cycling | Pregnant |
| 11-Jan-10 | PAB | BKR | IEA | AF | AF | Down | No | Yes [6] | No | No | No | Lactating | Lactating |
| 20-May-10 | PAB | NYB | AFR | AF | AF | Down | No | No | No | Yes | No | Pregnant | Pregnant |
| 20-May-10 | PAB | NYB | AFR | AF | AF | Down | No | No | No | Yes | No | Pregnant | Pregnant |
| 21-May-10 | PAB | MUK | NYB | AF | AF | Up | No | Yes [3] | No | No | Yes | Cycling | Pregnant |
| 26-Jul-10 | PAB | MAH | UMC | AF | AF | Up | No | Yes [1] | No | No | No | Cycling | Lactating |
| 16-Jul-10 | PAB | BKR | AFR | AF | AF | Up | NA | Yes [2] | No | No | No | Cycling | Pregnant |
| 16-Jul-10 | PAB | BKR | AFR | AF | AF | Up | NA | Yes [2] | No | No | No | Cycling | Pregnant |
| 10-Sep-10 | PAB | GUT | TMS | AF | SAF | Down | NA | No | No | NA |  | Pregnant | Cycling |
| 6-Oct-10 | PAB | MAH | AFR | AF | AF | Down | No | No | No | No | No | Cycling | Pregnant |
| 29-Oct-10 | PAB | GUT | NDW | AF | SAF | Down | No | No | No | No | No | Pregnant | Cycling |
| 23-Nov-10 | PAB | MAH | GUT | AF | AF | Down | No | Yes [5] | No | No | No | Pregnant | Pregnant |
| 24-Nov-10 | PAB | GUT | MUK | AF | AF | Up | No | Yes [6] | No | No | No | Pregnant | Cycling |
| 25-Nov-10 | PAB | AFR | BKR | AF | AF | Down | No | Yes [2] | No | No | No | Pregnant | Pregnant |
| 5-Dec-10 | PAB | ISU | TMS | SAF | SAF | Up | No | No | No | No | No | Cycling | Cycling |
| 27-Dec-10 | PAB | MAH | MUK | AF | AF | Down | No | Yes [1] | No | No | No | Pregnant | Cycling |
| 12-Jun-08 | BWE | MAG | NZE | AF | AF | Down | No | Yes [6] | No | No | No | Pregnant | Cycling |
| 22-Jul-08 | BWE | MAG | NZE | AF | AF | Down | No | Yes [6] | No | No | No | Pregnant | Cycling |
| 14-Aug-08 | BWE | MAG | FAI | AF | SAF | Down | No | Yes [33] | No | No | No | Pregnant | Cycling |
| 24-Aug-08 | BWE | MAG | NZE | AF | AF | Down | No | Yes [6] | No | No | No | Pregnant | Cycling |
| 19-Oct-08 | BWE | MAG | NZE | AF | AF | Down | No | Yes [6] | No | No | Yes | Pregnant | Cycling |
| 29-Oct-09 | BWE | MAG | KWR | AF | AF | Down | No | No | No | Yes | No | Pregnant | Cycling |
| 7-May-09 | BWE | MAG | KWR | AF | AF | Down | No | No | No | Yes | No | Cycling | Cycling |
| 23-Mar-09 | BWE | MAG | NZE | AF | AF | Down | No | Yes [6] | No | No | No | Cycling | Pregnant |
| 15-Mar-09 | BWE | MAG | NZE | AF | AF | Down | No | Yes [6] | No | No | No | Cycling | Pregnant |
| 3-Aug-09 | BWE | MAG | UNID | AF | AF | NA | No | NA | NA | No | NA | Pregnant | UNK |
| 29-Oct-09 | BWE | KWR | FAI | AF | AF | Down | No | Yes [3] | No | No | Yes | Cycling | Cycling |
| 1-Sep-09 | BWE | NZE | KWR | AF | AF | Up | No | Yes [11] | No | No | No | Pregnant | Cycling |
| 5-May-10 | BWE | NZE | FAI | AF | AF | Down | No | Yes [10] | No | No | No | Lactating | Cycling |
| 2-Jun-10 | BWE | FAI | MAG | AF | AF | Up | No | Yes [33] | No | No | No | Cycling | Lactating |
| 5-Aug-10 | BWE | MAG | NZE | AF | AF | Down | No | Yes [6] | No | No | No | Lactating | Lactating |
